# Feeding *Astragalus membranaceus* Root Improves the Rumen Fermentation Rate in Housed Goats through the Alteration of the Rumen Community Composition

**DOI:** 10.3390/microorganisms12061067

**Published:** 2024-05-25

**Authors:** Zhaoyu Peng, Mizuki Fujino, Mukul Anand, Yutaka Uyeno

**Affiliations:** 1 Graduate School of Medicine, Science and Technology, Shinshu University, Matsumoto 399-4598, Japan; 2 College of Veterinary Science and Animal Husbandry, DUVASU, Mathura 281001, India; drmukulanandvet@gmail.com

**Keywords:** carbohydrate digestion, enteric methane emission, microbial community analysis

## Abstract

Although *Astragalus membranaceus* root (AMR) has been noted as an ingredient in ruminant feed, the impacts of AMR feeding on rumen fermentation and the microbial community structure within the rumen are yet to be evaluated. This study investigated the effects of AMR supplementation on rumen fermentation characteristics and microbial community structures in goats. In two sets of feeding experiments, four Japanese native goats were fed AMR (10 g/kg DM/day/head) for three weeks per experiment. The rumen fluid samples were analyzed using high-performance liquid chromatography for fermentation products and next-generation sequencing for microbial analysis. The rumen fluid samples in the second experiment were also subject to an in vitro anaerobic fermentation test. The results indicated a significant modification, with a higher volatile fatty acid (VFA) content in the rumen fluid of goats in the feeding period than before feeding (*p* < 0.01). The microbial analysis revealed a significant increase in community diversity (*p* < 0.05) following AMR feeding, and the rumen bacterial community increased in two families belonging to the order Oscillospirales in Firmicutes (*p* < 0.05). The phylum Verrucomicrobiota was observed to be significantly less abundant after AMR feeding than during the control period (*p* < 0.05). Notably, the linear discriminant analysis revealed that the families with largely unknown functions in the rumen (Oscillospiraceae, Rikenellaceae, Muribaculaceae, and vadinBB97) were the determinants of the community split between control and AMR feeding. Increased fermentation rate by AMR feeding was also supported by an in vitro culture experiment, which resulted in faster VFA production without affecting methane production in total gas production. The study demonstrated that AMR can significantly facilitate change in the bacterial community structure in the goat rumen involving a shift of the favoring fibrolytic bacteria towards VFA production. The long-term effects of AMR supplementation and its applicability across different ruminant species, with potential benefits for animal health and productivity, should be addressed.

## 1. Introduction

Plants belonging to the *Astragalus* family have been extensively used as medicinal plants in East Asia for hundreds of years. Recent research has confirmed that *Astragalus* plants, particularly their roots, have a variety of therapeutic effects on issues such as inflammation, obesity, and cancer [1,2,3], and improve host animal health due to the high content of flavonoids, saponins, and other substances such as carbohydrates [4]. More recently, *Astragalus* has also been considered as a feed additive to improve animal health and farm animal production performance, achieving certain results in monogastric animals and ruminants [5,6,7,8,9].

Enteric fermentation in the rumen is systematically regulated by the inherent microbes in the organ, dependent on multiple factors such as feed composition and schedule, feeding behavior, age, and production stages, which further determine ruminant production efficiency and ruminant health status. In this context, several plant extracts and secondary plant metabolites were demonstrated to improve rumen fermentation by influencing the composition and activity of ruminal microorganisms. *A. membranaceus* root (AMR) appears to be an attractive ingredient because of its high bioactive components and its application as a new feed additive for modulating methane emissions is also worth exploring [10,11,12]. Although previous studies have applied ruminant feeding tests, for example, observing enhanced rumen fermentation achieved through moderate AMR usage [13], no evaluation report on AMR feeding has yet been published in terms of the linkage between rumen communities and fermentation properties. Therefore, the present study aimed to evaluate the effects of the incorporation of AMR into goat feed, focusing on relationships between the rumen fermentation and the alterations of their microbial community structure, which would link to the improvements.

## 2. Materials and Methods

### 2.1. Preparation of AMR

A commercially available, dried, and sliced AMR product (produced in China and purchased from a pharmacy in Ishikawa, Japan; Crude protein, 164 g/kg dry matter [DM]; Neutral detergent fiber [NDF], 630 g/kg DM; astragaloside IV, 750 mg/kg DM) was used for this study. The AMR was crushed using a mixer at the start of the experiment and passed through a 1 mm sieve for further use. 

### 2.2. Experiment Design, Feeding, and Sampling

All the animal experiments were conducted under the permission of the Animal Experiment Committee in accordance with the guidelines for animal experiments of Shinshu University (acceptance no. 022118). This study comprised two sets of intervention studies to fulfill the required sample number (*n* = 6), assumed by a power analysis for a two-sample paired-means test, with reference to the results from a previous study [13]. Following the first experiment performed from September to October (experiment 1), a replication study was conducted using the same animal sets and experimental design from February to March (experiment 2). The four goats used in the experiments (two 5-year-old dry females without conception and two 2-year-old castrated males) of the Japanese native breed (Shiba) were relatively similar in size (44 ± 3 kg in experiment 1 and 46 ± 4 kg in experiment 2). Two experiments were conducted in distinctively different climate conditions, based on the temperature range sourced by public reporting (e.g., highest temperature, average temperature, and lowest temperature, 27.5 °C, 17.3 °C, and 5.6 °C in experiment 1; 17.9 °C, 3.3 °C, and −9.2 °C in experiment 2).

Prior to AMR introduction in each experiment, rumen fluid samples were collected, referred to as PRE hereafter. Three weeks after the AMR introduction, rumen fluid samples were collected again, referred to as FEED. Excluding the addition of AMR to the feed, the feed ratios and management methods remained identical. 

The health status of the goats remained unchanged during the experiment without any symptoms of diarrhea or digestive disorder. The goats were housed in a paddock and fed twice daily at 0900 and 1600. The goats were originally fed basal ration, including the composition of the substrate comprising hay cubes and oat hay in a ratio of 2:3, as presented in Table 1, and were fed at a fixed amount of 500 g DM per animal per meal, accounting for 1 kg DM per day. The AMR was mixed into the feedstuff for three weeks at 1%DM, considered the optimal concentration from previous goat-feeding studies [5,13]. To determine daily feed intake, the amounts of remaining feed including AMR powder were recorded prior to the morning feeding of the following day. The rumen fluid was collected from the rumen of goats using a pump and an oral catheter before the morning feeding. The collected rumen fluid was flushed with CO_2_ gas immediately after collection and filtered through a cheesecloth. One part was stored at −20 °C for subsequent analysis.

### 2.3. In Vitro Fermentation Test

The in vitro fermentation test carried on in experiment 2 was performed based on the methods as per our previous paper [14]. A total of 60 mL of the filtered rumen fluid was diluted with McDougall’s buffer preheated at a ratio of 1:1. The diluted rumen fluid was flushed with N_2_ gas and transferred into a 250 mL sealed culture bottle (containing 1.2 g of substrate, crushed and milled with the same ration fed to the animals). The culture bottle was placed in a water bath at 40 °C for anaerobic culture at a stirring speed of 180 rpm. To avoid fermentation retardation by overpressure due to gas production by fermentation, a syringe was used to extract gas samples from the culture bottle (without breaking the airtight environment) at the two time points of 6 and 24 h during the culture period. The volume of the total gas produced during incubation was determined, followed by the analysis of the CH_4_ concentration in the gas to obtain the volume of in vitro CH_4_ production. For respective sampling timings (PRE and FEED), we conducted this incubation test again on the following day to check repeatability. The culture bottles and the rumen fluid samples were stored in the freezer of a refrigerator at −20 °C. 

### 2.4. Analysis of Rumen Fluids and the Cultivates

The parameters related to rumen fluid were analyzed as per the method described in our previous report [15]. The proportion of methane in the generated gas was analyzed using a gas chromatography system (Inorga LC Science Co., Ltd., Nara, Japan) with a steel column (Porapak Q 80/100 mesh 1/16 × 3 m, GL Sciences Inc., Tokyo, Japan) and a thermal conductivity detector (TCD). Helium was used as the carrier gas at a flow rate of 4 mL/min. A 0.2 mL aliquot of headspace gas was injected using a syringe. The volume of methane gas (in mL) was calculated using a standard gas mixture and the installed software SIC 480 II for ChromatoLogger (System Instruments Co., Ltd., Hachioji, Japan). 

The VFA content was analyzed through high-performance liquid chromatography using an LC-2000 system (JASCO Corporation, Tokyo, Japan) under the conditions of a previous study [16] as follows: column, Inertsil ODS-3 250 mm × 4.6 mm (GL Science Inc.); oven, 40 °C, mobile phase, 10% acetonitrile–0.02% perchloric acid; flow rate, 1 mL/min; detection, 210 nm absorbance. Operation control, peak detection, and quantification were performed using a software included in the system (ChromNAVI, JASCO Corporation, Tokyo, Japan). An assumption method based on the ruminal VFA stoichiometry equation was additionally employed to predict the rumen methane production from the animal [17,18]:

Predicted methane (mL/mol VFA) = 22.4 × (0.50 × AA − 0.25 × (PA + VA) + 0.50 × BA)

where AA, PA, BA, and VA are the production (mol% in total VFA) of acetate, propionate, butyrate, and valerate, and 22.4 is the gas volume (mL/mmol). 

The total bacteria and archaea were counted in the culture using real-time polymerase chain reaction (RT-PCR) [19]. DNA extraction was performed for the microbial analysis using the QIAamp DNA Stool Mini Kit (Qiagen, Hilden, Germany) following the manufacturer’s instructions, and stored at −20 °C until analysis. For the RT-PCR, the primer sets Eub338F (ACTCCTACGGGAGGCAG) and Eub522R (ACGTCRTCCMCNCCTTCCTC) were used for the total bacteria count, and qmcrA-F (TTCGGTGGATCDCARAGRGC) and qmcrA-R (GBARGTCGWAWCCGTAGAATCC) for prokaryotic archaea by quantifying a gene involved in methane production (mcrA) using the CFX96™ real-time system (Bio-Rad Inc., Hercules, CA, USA) and the SYBR(R) Premix Ex Taq™ Kit (Takara Bio Inc., Otsu, Japan). A total of 40 cycling conditions were performed; each cycle included denaturation at 95 °C for 10 s, annealing at 60 °C for 20 s, and extension at 72 °C for 30 s for 35 cycles, followed by a dissociation curve analysis to confirm the obtainment of the expected PCR end-products. The melting curve was analyzed to confirm that the expected PCR product was obtained. The extracted bacterial genomic DNA was subjected to 16S rRNA gene amplicon pyrosequencing. The primer set of 515F (5′-GTGCCAGCMGCCGCGGTAA-3′) and 806R (5′-GGACTACHVHHHTWTCTAAT-3′), the T100 thermal cycler (Bio-Rad Inc.), and Ex Taq (Takara Bio Inc.) were used to generate the amplicons. 

The thermal cycler conditions used for amplification were set as follows: initial denaturation at 95 °C for 10 s, and 25 cycles of 9 °C for 10 s, 57 °C for 30 s, and 62 °C for 30 s for the first PCR, whereas 10 cycles of 95 °C for 10 s, 57 °C for 30 s, and 62 °C for 30 s for the second PCR. The barcoded amplicons were subjected to paired-end sequencing on an Illumina MiSeq platform (Illumina, San Diego, CA, USA). The sequencing data were deposited in the DNA Data Bank of the Japan Sequence Read Archive (accession number: DRR510973-DRR510988). Post-sequencing processing was performed after filtering low-quality reads and trimming the adapter, barcode, and primer sequences using QIIME2. The reads from all the samples were clustered into amplicon sequence variants (ASVs) at a 97% sequence similarity level using the DADA2 platform. The alpha diversity (α-diversity) was measured using the Shannon index (Shannon *H*’), a way to measure the diversity of species in a community.

### 2.5. Statistical Treatment

The chemical parameters were obtained from both experiments 1 and 2 and analysis of variance (ANOVA) was applied for each measurement. The following model was used:

Y_ijk_ = μ + α_i_ + β_j_ + (αβ)_ij_ + γ_k_ + e_ijk_

where Y_ijk_ = observations for dependent variables; μ = overall mean; α_i_ = the fixed effect of experiments; β_j_ = the fixed effect of the sampling timing (PRE and FEED); (αβ)_ij_ = the interaction effect; γ_k_ = random effect of the animal; and e_ij_ = the residual error. Each animal was conceived as a random factor within the error term, considering all four animals were adults (i.e., ignorable level of weight gain), and were fed equal amounts of feed. In this model, it was also noted that the effects of treatment and time (within an experiment, 3 weeks) may be confounded. However, as the animals did not have any production measurement (growth or milk production), there was a limited range of changes in the rumen fermentation parameters in three weeks when compared with the differences (=error) among the animals. The application of this design was, therefore, selected. As stated, the in vitro culture test was performed only in experiment 2; therefore, the model construction was as follows:

Y_jk_ = μ + α_j_ + β_k_ + e_jk_.

The significance level was set at *p* < 0.05. Principal coordinate analysis (PCoA) was applied to the bacterial composition of all the sequenced samples to determine the beta diversity of the microbiota among the samples. All the statistical analyses were performed using the STATA BE 17 package (Stata Corp., College Station, TX, USA). The linear discriminant analysis effect size (LEfSe) was performed as described by Segata et al. [20]. 

## 3. Results

AMR did not change the amount of feed intake (1 kg DM/day) of the goats during the experimental period. Feeding AMR provided a relatively close fermentation pattern in the rumen in terms of the VFA profiles and archaeal proportion, as presented in Table 2, in which only the total VFA was high after the three weeks of AMR feeding compared with the pre-feeding period. After filtering the low-quality reads of the amplicon analysis, effective reads were further processed by denoising, filtering out chimeras, and removing the archaeal sequences. Finally, 119,273 ± 40,760 (mean ± SD) non-chimeric sequences were obtained. For all the samples, the number of amplicon sequencing variant (ASV) was 2624 in total, 1079 ± 269 on average. The analysis of the microbial community revealed a few distinct changes in the proportions of specific bacterial taxa (Table 3). Throughout the experiments, the phylum Bacteroidetes comprised the majority of the community. There was no significant change at the family level in the phylum, except for the family Muribacteriaceae, which tended to be higher in response to the AMR feeding. In the genus level, Prevotellaceae UCG-001 tended to decrease by the AMR feeding. After the AMR feeding, the rumen bacterial community increased in the families of Oscillospiraceae and its relative abundance in the uncultivated family of the order Oscillospirales (UCG-10) in Firmicutes. The phylum Verrucomicrobiota was observed to be significantly less abundant after the AMR feeding than during the control period, and the phyla Proteobacteria, Cyanobacteria, and Spirochaeota demonstrated a tendency to be lower after the feeding. The proportions of some groups changed across the two experiments; four taxa were lower in experiment 1—Succinivibrionaceae, Synegistaceae, Bacteroidales_RF16, and Lachnospira. Erysipelatoclostridiaceae in Firmicutes were higher in experiment 1 than in experiment 2. The interaction between experiment and treatment was determined only in the Firmicutes proportion.

**Figure 1:**
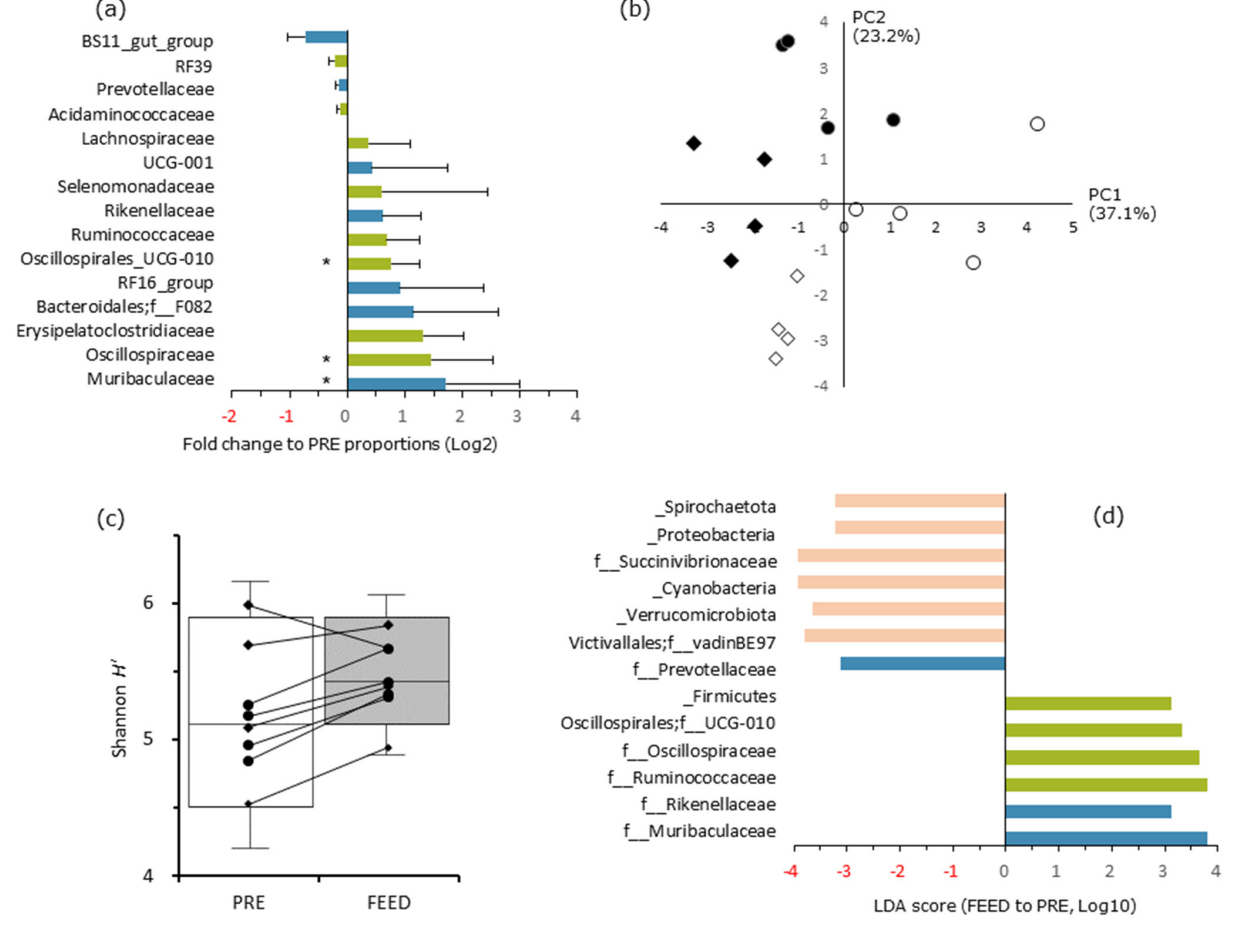
Transformed information about bacterial community structure among the samples. (**a**) The fold change in PRE to FEED periods in the abundance of distinct phylogenetic groups at a taxonomic level lower than the family level belonging to Bacteroidota (blue) and Firmicutes (green). Error bars indicate the standard error; an asterisk indicates *p* < 0.05. (**b**) The principal coordinate analysis of the samples at the operational taxonomic unit level using weighted Unifrac distance metric, with the samples labeled by sample attributions (◇ Expt 1-PRE; ◆, Expt 1-FEED; ○, Expt 2-PRE; ●, Expt 2-FEED). (**c**) The alpha diversity indices of the samples from the goats in experiment 1 (♦) and experiment 2 (●) indicated by Shannon *H’*. The higher the value, the higher the diversity of species in a particular community. A value of *H* = 0 indicates a community that only has one species. Data were shown in box plots; box, quartile interval; middle line, mean; whiskers, lower and upper limits of the 95% confidence level. As both distributions were found unequal, we employed the Wilcoxon signed rank test to compare the data between the PRE and FEED periods. (**d**) The LDA effect size (LEfSe) results display the proportional contrast of the phyla and families of the groups that differed significantly between PRE and FEED. Groups belonging to other phyla than Bacteroidota and Firmicutes were indicated in orange bars.

Detailed analyses revealed particular differences in the community structure between PRE and FEED (Figure 1). As presented in Figure 1a, considering the distributions of some representative taxonomic groups belonging to the two major phyla, Bacteroidetes and Firmicutes, the community alteration within phyla may have depended on the particular nature of the rumen of each animal. Regarding alpha diversity, the Shannon indices of the rumen bacterial community increased in the post-feeding period, and there was a significant difference in the distribution ranges (Figure 1b, *p* = 0.0499). Remarkable changes in the bacterial composition with regard to the AMR feeding were also observed in the PERMANOVA analysis of diversity (Figure 1c, *p* = 0.016) and LEfSe (Figure 1d). In the latter, Muribaculaceae (score 3.79) and Rikenellaceae (3.12) in Bacteroidota, Ruminococcaceae (3.79), Oscillospiraceae (3.66), and Oscillospirales were affected; f__UCG-010 (3.31) primarily affected the split, while a family in Verrucomicrobiota (vadinBE97, −3.75), phyla Cyanobacteria (−3.91), and Succinivibrionaceae in Proteobacteria (−3.93) were negatively affected. In addition, the PERMANOVA analysis also detected significant differences in the bacterial compositions between the experiments (*p* < 0.001). 

The in vitro rumen-simulated culture results in experiment 2 exhibited a significant increase in total gas production after 6 h of cultivation. However, the total gas production at 24 h, VFA production and its proportions, and methane production from the rumen liquor with or without the AMR feeding did not change (Table 4).

## 4. Discussion

The changes in the VFA content in rumen fluid before and after AMR feeding have been observed in a preceding report, in which the total VFA in the rumen of Tibetan sheep increased in response to AMR feeding at a level of 20 g/kg DM (2%) for 28 days [8]. Our testing condition was 10 g/kg for 21 d, indicating that the fermentation-promoting effect of AMR would depend on feeding conditions and animal variations. The increase in the VFA content in the rumen fluid post-AMR feeding may be related to its growth-promoting effect on animal production, as reported in a previous study [7,21]. Environmental biases, such as seasonal variation, may also explain the differences in the populations of some bacterial groups between experiments 1 and 2. Considering the tested goats were fed in low outside temperatures, they may need to adapt their rumen function to switch to harsh environmental conditions such as cold, heat, and drought [22]. However, the supplemental PCoA analysis determined that the effects of the AMR feeding exhibited consistent effects across the experiments. Since the interaction of the two effects (experiment and treatment) was only observed in the proportion of Firmicutes, the effects of the AMR feeding on the changes in the bacterial community structure are similar regardless of testing timing.

Wang et al. reported that the changes in the microbial community structure of the goat rumen in alpha diversity increase according to their growth, owing to the contribution of the thriving bacteria to fiber digestion efficiently [23]. Although this study used mature animals, the results largely follow the implications of enhanced nutrient acquisition by AMR feeding. Another study evaluated the effects of feeding AM powder consisting of stems and leaves to weaned piglets and found an increase in the diversity of the cecal bacterial community assessed by the alpha diversity of the community [6]. Our results were comparable, implying that *A. membranaceus* could be a substrate of particular bacteria groups inhabiting the animal gut. On the other hand, another study determined that an increased amount of AMR feeding (50 g/kg DM or more) conversely decreased the bacterial diversity in the rumen of Tibetan sheep [24]. 

Preceding in vivo studies have demonstrated a uniform increase in the proportion of Firmicutes by feeding AMR or a related substrate [7,24], as was determined in the current study. Regarding proportional changes in Bacteroidota, which increased in another assessment [25], only one group belonging to Bacteroidota (Muribacteria) changed with a significant decrease. This inconsistency may be attributed to the different test conditions, particularly the test materials, feed components, and amounts. The family-level groups that significantly changed were two closer groups belonging to Oscillospira and one family belonging to Verruccomicrobia, both of which are known rumen bacteria. However, most of these groups are yet to be cultured [26,27]. Their exact function and major roles in the rumen have, therefore, not yet been elucidated, even though both are known as carbohydrate utilizers [28,29]. As was also noticed in previous observations [28,30], the relative proportion of *Fibrobacteria* in the goat rumen was minor compared with cattle rumen. Fibrobacteroidetes comprised a low proportion of the rumen community of the test animals and were less affected by the dietary change in the current study. In contrast, Ruminococcaceae, a fibrolytic group in Firmicutes, increased in proportion in the rumen under the conditions of higher dietary fiber [30,31]. Further, the abundance of bacteria capable of processing a wide range of proteins and polysaccharides (referred to as versatile microbes) [32], such as *Prevotella* spp., appeared to decrease with feeding, as indicated in the LEfSe analysis.

While the relative abundance at the phylogenetic level of phyla and families in the lower-level distribution of the groups belonging to Firmicutes and Bacteroidetes may have changed with the AMR feeding, the study further sought to characterize the alterations in the rumen microbiota. Feeding AMR may not affect specific groups in the community, but to groups possessing similar functions of carbohydrate digestion, less abundant groups in the community may take important roles such as feed digestion. The results of community shifts designated by the fold changes in major bacteria groups, beta diversity, and discriminative changes illustrated by the LEfSe analysis could eliminate the effect on test animals. 

Compared to previous studies, the community dynamics analysis revealed an overall structural change in the goat rumen involving a shift from favoring fibrolytic bacteria towards VFA production facilitated by AMR. This alteration in the bacterial community structure in the rumen may be potentially attributed to the inclusion of various polysaccharides, some of which are considered functional in AMR [4]. These polysaccharides may exhibit several pharmacological activities such as immunomodulation, antioxidant, and antitumor activities [33]. In the present study, these polysaccharides could also serve as “functional substrates”, inducing rumen bacteria to enhance fermentation in some cases. Supplementary *A. membranaceus* to sheep feed has been speculated to increase the relative abundances of fiber-decomposing bacteria based on amplicon sequencing followed by gene function predictions [24]. However, feeding *A. membranacus*-derived material failed to improve the apparent digestibility of nutrients in weaned lambs [12].

Through the presented study, it was also conceived that the rumen methane production rate would change in an increasing or a decreasing manner due to feeding AMR. The methane production from the animal based on the ruminal VFA stoichiometry equation was additionally assumed in response to the changes in the rumen environments spiked by the AMR feeding, resulting in no observed change among the experiment blocks. In experiment 2, an in vitro cultivation experiment was conducted using rumen fluid taken from animals. This provided the fermentation results of increased gas production at an early stage (6 h) of cultivation. The total gas generated at 24 h and the methane production did not change regardless of AMR feeding. It may, therefore, be concluded that the AMR feeding would stimulate the rumen gas production without changing the overall methane production process. 

When considered together, the results suggest that the AMR supply may have several effects like increasing the activity of a wider range of bacteria related to carbohydrate digestion without changing the production rate of wastes, including methane. AMR treatment would spike fermentation pathways in ruminant nutritional uptake and metabolism to shift in a better direction. These changes finally affected the increase in in vivo ruminal VFA concentration and in vitro gas production rate which reflected improved fermentation efficiency. On the other hand, some uncertainties have been left unanswered: (1) while two experiments were conducted under limited feeding conditions, different experimental settings such as ad libitum feeding would have affected the efficiency of dry matter intake, growth performance, and rumen methane production in a more contrasting way, followingly; (2) which components in AMR affected the structure of the rumen microbial community and the functions of polyphenols, carbohydrates, or other nutrients; and (3) how the increase in ruminal VFA production would further induce any effect on host physiology. These new hypotheses indicate improvements in both nutrition availability and animal performance. It is further speculated that AMR may enhance the stress resistance of the gastrointestinal microbial flora in experimental animals, which may also be related to the ability of AMR to enhance the immunity and nutrition uptake of the animal [9,13]. This would also be feasible in the changes reflecting improvements in nutrition availability which has already been evidenced by preceding studies using monogastric animals [34]. Future research should focus on the long-term effects of AMR supplementation and explore its applicability across different ruminant species and on the mechanistic basis of how AMR influences microbial ecosystems within the rumen, especially in lactation cows. In addition, if a specific type of carbohydrate can be determined to enhance uncultivated microbes in the rumen, it would be a worthy supplement for the enrichment of the strains to isolate and explore the characteristics.

## 5. Conclusions

This study revealed that the inclusion of AMR for three weeks resulted in an increased total VFA concentration in goat rumen. The results highlight AMR as a promising feed additive that improves rumen fermentation and alters the microbial community composition in goats, particularly by increasing relatively minor groups who are yet-to-be-cultured members. Moreover, the community shift is likely attributed to the variations in polysaccharides in AMR, suggesting that AMR may confer certain benefits for the rumen by redirecting fermentation pathways towards increased VFA production. These changes are beneficial for enhancing feed efficiency, aligning with sustainable livestock management goals.

## Figures and Tables

**Table 1:** Ration nutrients.

		Oat Hay	Alfalfa Hay Cube
Dry matter (DM)	g/kg	903	922
Crude protein	g/kg DM	90	205
Ether extract	g/kg DM	21	24
Ash	g/kg DM	60	100
Neutral detergent fiber (amylase-treated)	g/kg DM	518	425
Acid detergent fiber	g/kg DM	307	319
Metabolizable energy	MJ/kg	12.0	10.9

The ration consisted of 300 g oat hay and 200 g of alfalfa hay cube per meal per head.

**Table 2:** Properties of the rumen fluids.

	Experiment 1		Experiment 2		SE	*p*		
	PRE	FEED	PRE	FEED		Experiment	Treatment	E × T
Total VFA (mmol/L)	66.1	97.3	80.3	111.5	5.9	0.254	0.008	0.998
Acetate (mol%)	64.4	64.8	64.9	64.6	0.5	0.935	0.967	0.767
Propionate (mol%)	21.1	20.6	22.0	23.1	0.6	0.273	0.586	0.514
Butyrate (mol%)	14.4	14.6	13.1	12.3	0.5	0.100	0.683	0.623
Archaea (% to total bacteria)	2.7	2.9	3.4	2.4	0.0	0.907	0.568	0.374
Estimated methane (mL/mmol VFA)	7.6	7.7	7.5	7.3	0.1	0.178	0.816	0.491

E × T, interaction of experiment and treatment; VFA, volatile fatty acid. In all the samples, valerate was detected <1% of the total VFA.

**Table 3:** Bacterial community analysis of rumen fluid.

	Experiment 1		Experiment 2		SE		*p*	
	PRE	FEED	PRE	FEED		Experiment	Treatment	E × T
Bacteroidota	0.782	0.747	0.696	0.736	0.036	0.107	0.904	0.075
f__Prevotellaceae	0.569	0.495	0.506	0.476	0.063	0.222	0.131	0.489
g__*Prevotella*	0.447	0.417	0.434	0.404	0.008	0.590	0.447	0.997
g__Prevotellaceae UCG-001	0.025	0.018	0.011	0.009	0.003	0.109	0.025	0.733
g__Prevotellaceae UCG-003	0.018	0.018	0.024	0.017	0.001	0.566	0.518	0.487
f__Rikenellaceae	0.105	0.136	0.064	0.099	0.037	0.061	0.103	0.927
(g__Rikenellaceae RC9 gut group)								
f__Bacteroidales_RF16_group	0.037	0.040	0.063	0.057	0.010	0.002	0.768	0.362
(g__Bacteroidales_RF16_group)								
f__F082 (g_F082)	0.051	0.049	0.021	0.057	0.025	0.378	0.196	0.153
f__Bacteroidales_BS11_gut_group	0.018	0.020	0.026	0.013	0.010	0.941	0.282	0.155
(g__ Bacteroidales_BS11_gut_group)								
f__Muribaculaceae	0.003	0.005	0.004	0.025	0.011	0.076	0.061	0.112
Firmicutes	0.131	0.184	0.157	0.159	0.022	0.912	0.034	0.047
f__Lachnospiraceae (g__*Butyrivibrio*)	0.021	0.023	0.039	0.041	0.010	0.005	0.701	0.979
f__Oscillospiraceae	0.010	0.025	0.018	0.022	0.008	0.507	0.038	0.229
f__Selenomonadaceae (g__*Selenomonas*)	0.003	0.004	0.017	0.016	0.015	0.103	0.999	0.894
f__Erysipelatoclostridiaceae (g__UCG004)	0.017	0.040	0.011	0.009	0.012	0.001	0.100	0.058
f__Bacilli RF39	0.014	0.010	0.011	0.010	0.005	0.649	0.371	0.587
f__ Oscillospirales UCG-010	0.009	0.016	0.008	0.009	0.003	0.521	0.027	0.080
f__Acidaminococcaceae	0.006	0.006	0.007	0.004	0.005	0.718	0.513	0.574
(g__*Succiniclasticum*)								
f__Ruminococcaceae	0.003	0.005	0.007	0.009	0.003	0.052	0.169	0.772
Verrucomicrobiota	0.030	0.018	0.024	0.021	0.007	0.537	0.047	0.243
f__WCHB1-41	0.006	0.006	0.010	0.007	0.004	0.253	0.470	0.538
f__ Victivallales vadinBE97	0.017	0.010	0.011	0.007	0.005	0.099	0.061	0.534
Synergistota (f__Synergistaceae)	0.005	0.006	0.029	0.021	0.009	0.002	0.463	0.325
Proteobacteria	0.003	0.002	0.029	0.014	0.008	0.002	0.094	0.094
f__Succinivibrionaceae	0.001	0.000	0.023	0.010	0.007	0.001	0.072	0.082
Cyanobacteria (f__Gastranaerophilales)	0.019	0.012	0.026	0.019	0.007	0.065	0.057	0.912
Spirochaetota (f__Spirochaetaceae)	0.007	0.005	0.008	0.006	0.002	0.051	0.073	0.777
Fibrobacterota (g__*Fibrobacter*)	0.002	0.003	0.006	0.003	0.002	0.843	0.531	0.226

Taxa more than 0.5% (family and genus level) or 0.2% (phylum level) of the total proportion on average are listed in the table. The taxon between parentheses indicates only one genus or family determined in the upper clade (family or phylum). E × T, interaction of experiment and treatment.

**Table 4:** In vitro fermentation results (experiment 2).

		PRE		FEED		*p*
Fermentation properties in the gas phase						
GP 6 h	mL	68	±5	74	±4	0.025
GP 24 h	mL	120	±4	123	±7	0.596
Methane% 6 h	%	9.8	±1.5	8.3	±3.7	0.516
Methane% 24 h	%	15.6	±1.5	14.2	±2.7	0.171
MP 6 h	mL	6.6	±0.7	6.2	±2.0	0.869
MP 24 h	mL	18.7	±0.4	17.5	±1.5	0.507
Fermentation properties in the liquid phase of 24 h culture						
Total VFA	mmol/L	84.1	±6.1	98.9	±7.3	0.092
Acetate	mol%	65.9	±1.7	66.3	±2.0	0.739
Propionate	mol%	22.1	±1.4	22.6	±1.5	0.286
Butyrate	mol%	12.0	±1.0	11.2	±1.1	0.447

GP, gas production; MP, methane production. Data are expressed as mean ± SD.

## Data Availability

The sequencing data presented in this study were deposited in the DNA Data Bank of the Japan Sequence Read Archive [https://www.ddbj.nig.ac.jp/index.html; accessed on 1 May 2024] (accession Number: DRR510973-DRR510988).

## References

[1] Nie T., Zhao S., Mao L., Yang Y., Sun W., Lin X., Liu S., Li K., Sun Y., Li P. (2018). The natural compound, formononetin, extracted from *Astragalus membranaceus* increases adipocyte thermogenesis by modulating PPARγ activity. Brit. J. Pharmacol..

[2] Zheng Q., Zhuang Z., Wang Z.-H., Deng L.-H., Jin W.-J., Huang Z.-J., Zheng G.-Q., Wang Y. (2020). Clinical and preclinical systematic review of *Astragalus membranaceus* for viral myocarditis. Oxidat. Med. Cell. Biol..

[3] Auyeung K.K., Han Q.-B., Ko J.K. (2016). Astragalus membranaceus: A review of its protection against inflammation and gastrointestinal cancers. Am. J. Chin. Med..

[4] Jin M., Zhao K., Huang Q., Shang P. (2014). Structural features and biological activities of the polysaccharides from *Astragalus membranaceus*. Int. J. Bio. Macromol..

[5] Hao X., Wang P., Ren Y., Liu G., Zhang J., Leury B., Zhang C. (2020). Effects of *Astragalus membranaceus* roots supplementation on growth performance, serum antioxidant and immune response in finishing lambs. Asian-Aust. J. Anim. Sci..

[6] Che D., Adams S., Wei C., Gui-Xin Q., Atiba E.M., Hailong J. (2019). Effects of *Astragalus membranaceus* fiber on growth performance, nutrient digestibility, microbial composition, VFA production, gut pH, and immunity of weaned pigs. MicrobiologyOpen.

[7] Wei H., Ding L., Wang X., Yan Q., Jiang C., Hu C., Wang G., Zhou Y., Henkin Z., Degen A.A. (2021). *Astragalus* root extract improved average daily gain, immunity, antioxidant status and ruminal microbiota of early weaned yak calves. J. Sci. Food Agric..

[8] Abdallah A., Zhang P., Elemba E., Zhong Q., Sun Z. (2020). Carcass characteristics, meat quality, and functional compound deposition in sheep fed diets supplemented with *Astragalus membranaceus* by-product. Anim. Feed Sci. Technol..

[9] Shao P., Sha Y., Liu X., He Y., Wang F., Hu J., Wang J., Li S., Chen X., Yang W. (2024). Supplementation with *Astragalus* root powder promotes rumen microbiota density and metabolome interactions in lambs. Animals.

[10] Islam M., Kim S.-H., Mamuad L.L., Yu Z., Lee S.-S. (2021). Holstein and Jersey steers differ in rumen microbiota and enteric methane emissions even fed the same total mixed ration. Front. Microbiol..

[11] Min B.R., Solaiman S., Waldrip H.M., Parker D., Todd R.W., Brauer D. (2020). Dietary mitigation of enteric methane emissions from ruminants: A review of plant tannin mitigation options. Anim. Nutr..

[12] Zhong R.Z., Yu M., Liu H.W., Sun H.X., Cao Y., Zhou D.W. (2012). Effects of dietary Astragalus polysaccharide and *Astragalus membranaceus* root supplementation on growth performance, rumen fermentation, immune responses, and antioxidant status of lambs. Anim. Feed Sci. Technol..

[13] Wang X., Ding L., Wei H., Jiang C., Yan Q., Hu C., Jia G., Zhou Y., Henkin Z., Degen A. (2021). *Astragalus membranaceus* root supplementation improves average daily gain, rumen fermentation, serum immunity and antioxidant indices of Tibetan sheep. Animal.

[14] Abdelazeem S., Takeda K.-i., Kurosu K., Uyeno Y. (2020). Fermentative quality and animal acceptability of ensiled persimmon skin with absorbents for practical use in ruminant feed. Animals.

[15] Mousa S.A., Malik P.K., Kolte A.P.K.P., Bhatta R., Kasuga S., Uyeno Y. (2019). Evaluation of in vitro ruminal fermentation of ensiled fruit byproducts and their potential for feed use. Asian-Australas. J. Anim. Sci..

[16] Takeuchi K., Takeuchi M., Kakino W., Uyeno Y. (2022). Biofilm bacterial dynamics and changes in inorganic nitrogen density due to the presence of freshwater pearl mussels. mSphere.

[17] Wolin M.J. (1960). A Theoretical rumen fermentation balance. J. Dairy Sci..

[18] Ramin M., Huhtanen P. (2012). Development of an in vitro method for determination of methane production kinetics using a fully automated in vitro gas system—A modelling approach. Anim. Feed Sci. Technol..

[19] Kido K., Tejima S., Haramiishi M., Uyeno Y., Ide Y., Kurosu K., Kushibiki S. (2019). Provision of beta-glucan prebiotics (cellooligosaccharides and kraft pulp) to calves from pre-to post-weaning period on pasture. Anim. Sci. J..

[20] Segata N., Izard J., Waldron L., Gevers D., Miropolsky L., Garrett W.S., Huttenhower C. (2011). Metagenomic biomarker discovery and explanation. Genome Biol..

[21] Jiang C., Ding L., Dong Q., Wang X., Wei H., Hu C., Ma C., Yan Q., Zhou Y., Degen A.A. (2021). Effects of root extracts of three traditional Chinese herbs as dietary supplements on dry matter intake, average daily gain, rumen fermentation and ruminal microbiota in early weaned yak calves. Anim. Feed Sci. Technol..

[22] Giger-Reverdin S., Domange C., Broudiscou L.P., Sauvant D., Berthelot V. (2020). Rumen function in goats, an example of adaptive capacity. J. Dairy Res..

[23] Wang L., Xu Q., Kong F., Yang Y., Wu D., Mishra S., Li Y. (2016). Exploring the goat rumen microbiome from seven days to two years. PLoS ONE.

[24] Wang X., Hu C., Ding L., Tang Y., Wei H., Jiang C., Yan Q., Dong Q., Degen A.A. (2021). *Astragalus membranaceus* alters rumen bacteria to enhance fiber digestion, improves antioxidant capacity and immunity indices of small intestinal mucosa, and enhances liver metabolites for energy synthesis in tibetan sheep. Animals.

[25] Shao P., Sha Y., Liu X., He Y., Guo X., Hu J., Wang J., Li S., Zhu C., Chen G. (2023). *Astragalus* additive in feed improved serum immune function, rumen fermentation and the microbiota structure of early-weaned lambs. J. Appl. Microbiol..

[26] Zhao Z.W., Ma Z.Y., Wang H.C., Zhang C.F. (2021). Effects of rumen-protected methionine and lysine supplementation on milk yields and components, rumen fermentation, and the rumen microbiome in lactating yaks (*Bos grunniens*). Anim. Feed Sci. Technol..

[27] Wallace R.J., Sasson G., Garnsworthy P.C., Tapio I., Gregson E., Bani P., Huhtanen P., Bayat A.R., Strozzi F., Biscarini F. (2019). A heritable subset of the core rumen microbiome dictates dairy cow productivity and emissions. Sci. Adv..

[28] Wang L., Wu D., Yan T., Wang L. (2018). The impact of rumen cannulation on the microbial community of goat rumens as measured using 16S rRNA high-throughput sequencing. J. Anim. Physiol. Anim. Nutr..

[29] Mackie R.I., Aminov R.I., Hu W., Klieve A.V., Ouwerkerk D., Sundset M.A., Kamagata Y. (2003). Ecology of uncultivated *Oscillospira* species in the rumen of cattle, sheep, and reindeer as assessed by microscopy and molecular approaches. Appl. Environ. Microbiol..

[30] Liu K., Xu Q., Wang L., Wang J., Guo W., Zhou M. (2017). The impact of diet on the composition and relative abundance of rumen microbes in goat. Asian-Aust. J. Anim. Sci..

[31] Xue B., Wu M., Yue S., Hu A., Li X., Hong Q., Wang Z., Wang L., Peng Q., Xue B. (2022). Changes in rumen bacterial community induced by the dietary physically effective neutral detergent fiber levels in goat diets. Front. Microbiol..

[32] Betancur-Murillo C.L., Aguilar-Marín S.B., Jovel J. (2022). Prevotella: A key player in ruminal metabolism. Microorganisms.

[33] Luo Y., Su L., Su R., Wang B., Liu C., Wang Z., Zhao L., Jin Y. (2020). Effects of *Astragalus membranaceus* supplementation on oxidative stability of Cashmere goat. Food Sci. Nutr..

[34] Guo L., Hua J., Luan Z., Xue P., Zhou S., Wang X., Qin N. (2019). Effects of the stems and leaves of *Astragalus membranaceus* on growth performance, immunological parameters, antioxidant status, and intestinal bacteria of quail. Anim. Sci. J..

